# 17-hydroxyprogesterone quantification in dried blood spots by high performance liquid chromatography-tandem mass spectroscopy

**DOI:** 10.1186/1687-9856-2015-S1-P44

**Published:** 2015-04-28

**Authors:** Kate Waller, Michael Henman, Tony Huynh, Janet Warner

**Affiliations:** 1 Mater Pathology, Brisbane, QLD, Australia; 2 The Canberra Hospital, Canberra, ACT, Australia

## 

Measurement of 17-hydroxyprogesterone (17-OHP) in daily dried blood spot profiles via radioimmunoassay is a convenient and accepted method for monitoring of glucocorticoid therapy in Congenital Adrenal Hyperplasia. Characteristics of this method that serve to limit its clinical usefulness include its lack of specificity for 17-OHP, and a relatively high limit of detection. Mass Spectroscopy is the gold standard method for steroid quantification. We aim to establish a High Performance Liquid Chromatography-Tandem Mass Spectroscopy (HPLC-MS/MS) method for dried blood spot 17-OHP quantification and develop normative age- and tanner-specific, as well as Congenital Adrenal Hyperplasia genotype-phenotype correlated reference ranges to guide glucocorticoid therapy.

Four 3mm blood spots were punched from patient dried blood spot filter paper specimens. 17-OHP was eluted into solvent and concentrated using liquid nitrogen. Steroids were separated using high performance liquid chromatography and quantitated by Tandem Mass Spectrometry. For the radioimmunassay / HPLC-MS/MS correlations, measurements were performed by both methods on 49 samples from children with glucocorticoid-dependent Congenital Adrenal Hyperplasia, as well as children undergoing dynamic endocrine function testing at The Mater Children’s Hospital. Reference samples for HPLC-MS/MS calibration, and determination of sensitivity, precision, and recovery were prepared using whole blood samples spiked with 17-OHP spotted onto filter paper. Concentrations were expressed as mean (nmol/L), standard deviation, and coefficient of variation (%).

There was excellent correlation between HPLC-MS/MS and radioimmunoassay methods (r^2^=0.9610). For the radioimmunoassay method, the lower limit of quantification has been established at <5 nmol/L while LC-MS/MS allows detection to 1.0 nmol/L (1.2nmol/L, 0.26, 22%) with a proposed lower limit of quantification of 1.5 nmol/L (1.3 nmol/L, 1.3, 13%).

We have established an accurate, reliable, and specific method for quantifying 17-OHP concentrations from dried blood spot using HPLC-MS/MS. This will allow for the establishment of clinically relevant and time-specific reference ranges to guide glucocorticoid dose adjustment in children with Congenital Adrenal Hyperplasia.

